# Comparison of agreement in asthmagen exposure assessments between rule-based automatic algorithms and a job exposure matrix in healthcare workers in Australia and Bhutan

**DOI:** 10.1186/s12889-022-14514-w

**Published:** 2022-11-16

**Authors:** Rajni Rai, Lin Fritschi, Deborah C Glass, Nidup Dorji, Sonia El-Zaemey

**Affiliations:** 1 School of Population Health, Curtin University, Kent St, 6102 Bentley, WA Australia; 2 School of Public Health and Preventive Medicine, Monash University, Melbourne, VIC Australia; 3 Faculty of Nursing and Public Health, Khesar Gyalpo University of Medical Sciences of Bhutan, Thimphu, Bhutan

**Keywords:** Occupational exposure assessment, Epidemiology methodology, Asthma, Healthcare workers

## Abstract

**Background:**

Assessment of occupational exposures is an integral component of population-based studies investigating the epidemiology of occupational diseases. However, all the available methods for exposure assessment have been developed, tested and used in high-income countries. Except for a few studies examining pesticide exposures, there is limited research on whether these methods are appropriate for assessing exposure in LMICs. The aim of this study is to compare a task-specific algorithm-based method (OccIDEAS) to a job-specific matrix method (OAsJEM) in the assessment of asthmagen exposures among healthcare workers in a high-income country and a low- and middle- income country (LMIC) to determine an appropriate assessment method for use in LMICs for future research.

**Methods:**

Data were obtained from a national cross-sectional survey of occupational asthmagens exposure in Australia and a cross-sectional survey of occupational chemical exposure among Bhutanese healthcare workers. Exposure was assessed using OccIDEAS and the OAsJEM. Prevalence of exposure to asthmagens and inter-rater agreement were calculated.

**Results:**

In Australia, the prevalence was higher for a majority of agents when assessed by OccIDEAS than by the OAsJEM (13 versus 3). OccIDEAS identified exposures to a greater number of agents (16 versus 7). The agreement as indicated by κ (Cohen’s Kappa coefficient) for six of the seven agents assessed was poor to fair (0.02 to 0.37). In Bhutan, the prevalence of exposure assessed by OccIDEAS was higher for four of the seven agents and κ was poor for all the four agents assessed (-0.06 to 0.13). The OAsJEM overestimated exposures to high-level disinfectants by assigning exposures to all participants from 10 (Bhutan) and 12 (Australia) ISCO-88 codes; whereas OccIDEAS assigned exposures to varying proportions of participants from these ISCO-codes.

**Conclusion:**

There was poor to fair agreement in the assessment of asthmagen exposure in healthcare workers between the two methods. The OAsJEM overestimated the prevalence of certain exposures. As compared to the OAsJEM, OccIDEAS appeared to be more appropriate for evaluating cross-country exposures to asthmagens in healthcare workers due to its inherent quality of assessing task-based determinants and its versatility in being adaptable for use in different countries with different exposure circumstances.

## Introduction

Assessment of occupational exposures is an integral component of population-based studies investigating the epidemiology of occupational diseases. However, exposure assessment in large population-based studies is often difficult because direct measurements of exposures are usually not feasible [1]. There are several methods to retrospectively assess occupational exposures such as self-reports, job exposure matrices (JEMs), and expert opinion, each with their own advantages and limitations. Self-reports of exposures are easy but are time consuming to apply and code in large studies, and are prone to misclassification [2]. JEMs can be easily and rapidly applied in large studies, saving time and resources but may result in misclassification because the same exposure is assigned to all workers in a job without taking into account inter-individual variations [1, 3]. Assessment by an expert is considered to be the most credible way to assess exposures [3], but using this method in large studies can be expensive and time consuming. Multiple experts may be required if the study involves a wide range of agents. In addition, the decision rules to assign exposures may not be explicit and may vary from one expert to another limiting the reproducibility of assessments [4]. In recent years, newer methods have been developed for exposure assessment such as the use of automated algorithms derived from experts and JEMs where exposure estimates are derived from historical exposure measurements [3]. There is some evidence indicating that these methods are as reliable as the traditional methods [5].

Exposures in low- and middle- income countries (LMICs) are likely to be different from those in high-income countries because of differences in legislation, work environments, raw materials and the availability of control measures. Previous studies have shown that many control measures and safer alternatives are not available in LMICs [6, 7]. In addition, there can be weak enforcement of regulations, low workplace safety culture, and a lack of local occupational health and safety expertise [8, 9]. All the available methods for hazardous exposure assessment have been developed, tested and used in high-income countries. Except for a few studies examining pesticide exposures [10, 11], there is limited research on whether these methods are appropriate for assessing exposure in LMICs. The aim of this study is to compare two methods of exposure assessments, a task-specific algorithm-based method (OccIDEAS) and a job-specific matrix method (OAsJEM), in the assessment of asthmagen exposures among healthcare workers in a high-income country and a LMIC to determine an appropriate assessment method for use in LMICs for future research.

OccIDEAS is a web application based on the expert assessment method that automatically assesses many occupational exposures [12]. It comprises of job modules, which are a set of questionnaires for a specific occupation. These job modules contain task modules with questions on particular tasks (e.g., sterilising instruments, using x-rays) that are linked to agents associated with the task. Using responses to the questions in the modules, algorithms developed by experts, automatically assign the probability of exposures. OccIDEAS has been used in three large studies in Australian workers to estimate the prevalence of exposure to carcinogens, asthmagens, and to noise and ototoxic agents [13–15]. It has also been used in breast cancer epidemiological studies to assess exposure to solvents [16], radiation [17], engine exhausts [18], and pesticides [19]; and for exposure to asbestos for a mesothelioma registry [20]. OccIDEAS assesses exposure to 27 asthmagen groups, which include a comprehensive list of agents that are known to be asthmagens and are used in occupational settings in Australia [21].

The occupational asthma-specific JEM (OAsJEM: https://oasjem.vjf.inserm.fr/home-OAsJEM/) is a recently updated version of an asthma-specific JEM developed in the late 1990s [22]. The original JEM, developed in France and known as the SK-JEM, assessed exposures (exposed/unexposed) to 18 asthmagens and four agents with low risk for asthma and has been used in several large epidemiological studies [23, 24]. The updated version assesses exposures to 30 asthmagen agents and classifies exposures semi-quantitatively into three groups: i) high: high probability of exposure and moderate to high intensity, ii) medium: low to moderate probability or low intensity of exposure, such as ‘high probability and low intensity’ or ‘low probability and moderate to high intensity, and iii) no exposure: unlikely to be exposed (low probability and low intensity) [22]. The updated version has been used in epidemiological studies conducted in Northern Europe, Australia and Denmark [25–27].

## Methods

### Study population and data collection

The present study uses data from two sources: (1) The Australian Work Exposure Study-Asthma (AWES-Asthma), and (2) a cross-sectional study conducted in Bhutan (hereafter referred to as the Bhutan healthcare workers study).

### The AWES-Asthma study

This study was a cross-sectional telephone survey conducted in 2014 to examine the prevalence of occupational exposure to asthmagens among Australian workers ^14^. Participants were randomly selected from a list of Australian households provided by a commercial broker. All Australian residents aged 18 to 64 years currently in paid employment who were able to complete an interview in English were eligible for the study. A total of 38,051 households were contacted, of which 6314 were eligible for the study and 4878 participants completed the interviews. Participants were asked to provide demographic information such as age, gender, education, country of birth and postcode. They were then asked about their job and the main tasks they performed in that job. The present study uses data from 426 healthcare workers in the AWES-Asthma study.

### The Bhutan healthcare workers study

This was a cross-sectional study carried out in 2019 among healthcare workers from three public hospitals in the western region of Bhutan to assess occupational exposures to hazardous chemicals [28]. All healthcare workers aged 18 to 60 years who were working in the three study hospitals were eligible to participate. Data were collected using hard-copy questionnaires distributed to healthcare workers who were randomly selected from the three hospitals. A total of 384 questionnaires were distributed, of which 370 were completed. These questionnaires collected demographic information such as age, gender, education, and information on the participants’ current job such as job title, hospital they were working in and how long they had worked as a healthcare worker. The questionnaire also obtained information on the tasks carried out in their jobs (e.g., suturing, working with chemicals in the laboratory, cleaning and sterilisation) and about any control measures used (e.g., fume hoods, personal protective equipment).

### Grouping of agents for the analyses

Because OccIDEAS assesses exposures to 27 asthmagen groups (refer to the paper by Crewe et al., 2016 [21] for the list of asthmagens) and the OAsJEM assesses exposures to 30 agents, some agents were grouped together for the analyses in this study and some were dropped (Table 1). This resulted in the assessment of exposure to a total of 19 agents for the AWES-Asthma study and nine agents for the Bhutan healthcare workers study.

**Table 1:** Grouping of agents assessed in OccIDEAS and the Asthma-specific JEM (OAsJEM) for the analyses

No.	OAsJEM agents	No.	OccIDEAS agents	No.	Agents for the present study
1.	Animals	1.	Derived from animals	1.	Animals
2.	Fish/shellfish	2.	Derived from fish/shellfish	2.	Fish
3.	Flour	3.	Flour	3.	Flour
4.	Foods	4.	Foods	4.	Foods
5.	Plant-related dusts	5.	Flowers	5.	Plants
		6.	Derived from plants-other		
6.	Enzymes	7.	Biological enzymes	7.	Enzymes
7.	House dust mites	8.	Arthropods or mites	6.	Mites
8.	Storage mites				
9.	Plant mites				
10.	Latex	9.	Latex*	8.	Latex
11.	Moulds	10.	Bio aerosols* (Alternaria, Chrysonilia sitophilia, Neurospora, Penicillium)	9.	Bio aerosols
12.	Endotoxins				
13.	Drugs	11.	Medications*	10.	Drugs
14.	High-level disinfectants	12.	Aldehydes*	11.	High-level disinfectants
		13.	Ethylene oxide*		
15.	Aliphatic amines	14.	Amines*	12.	Amines
16.	Isocyanates	15.	Isocyanates*	13.	Isocyanates
17.	Acrylates	16.	Acrylates*	14.	Acrylates
18.	Epoxy resins	17.	Epoxy*	15.	Epoxy
19.	Wood	18.	Wood dusts	16.	Wood
20.	Metals	19.	Metals	17.	Metals
		20.	Soldering		
21.	Herbicides	21.	Pesticides	18.	Pesticides
22.	Insecticides				
23.	Fungicides				
24.	Indoor cleaning + Bleach	22.	Industrial cleaning /sterilising agents* + Ammonia*	19.	Cleaning agents
25.		23.			
26.	Textile		-		dropped
27.	Persulphates/henna		-		dropped
28.	Metal working fluids		-		dropped
29.	Organic solvents		-		dropped
30.	Exhaust fumes		-		dropped
	-	24.	Acids*		dropped
	-	25.	Anhydrides		dropped
	-	26.	Other reactive chemicals*		dropped
	-	27.	Reactive dyes*		dropped

*OccIDEAS agents assessed in the Bhutan healthcare workers study. All 27 OccIDEAS agents were assessed in the AWES-Asthma study

### Exposure assessment by OccIDEAS

#### The AWES-Asthma study

Basic information on the participants’ current job was collected and 426 health care workers were identified. The job modules assigned were for healthcare professionals and laboratory workers, as well as cleaners and office workers who worked in healthcare settings. These modules comprised of questions on tasks they performed in their job where there was possible asthmagen exposure and on control measures where appropriate. Tasks included working in the laboratory (e.g., the use of various laboratory chemicals), in dental and general surgery (e.g., applying antiseptics, using diathermy), or during sterilisation and general cleaning tasks. These questions in the job modules examined exposure to 27 asthmagen groups (Table 1). For exposure assessment, predetermined algorithms with rules based on expert opinion and evidence from literature were applied to determine the probability of exposure (no/probable/possible). The ‘possible’ exposures were then reviewed by project staff and assigned as ‘probable’ or ‘no’ exposure using all available information.

#### The Bhutan healthcare workers study

The data from the 370 questionnaires were entered into OccIDEAS. As described above, predetermined algorithms were then applied to the responses to the questions to determine the likelihood of exposure (no/probable/possible) and the ‘possible’ exposures were then manually recoded as ‘probable’ or ‘no’ exposure using the available information. Exposures were assessed for 14 asthmagen groups known to be used in the healthcare sector in Bhutan (Table 1).

### Exposure assessment using the OAsJEM

For both studies, participants’ jobs were first coded according to the ISCO-88 four digit coding system using information on job titles, main tasks performed, education and employer industry. As per the JEM instructions, the OAsJEM was then applied in two steps. In the first step, the OAsJEM was applied to all ISCO-88 groups in both studies and exposures were assigned as high/medium/no exposures. For the second step, the exposures in certain ISCO-88 groups (mainly those with heterogeneous exposures) for both studies were re-evaluated by RR and further reviewed by LF and S-EZ, and a new exposure level was assigned based on additional available information about the job and tasks (Table 2). For example, for all nursing and midwifery professionals (ISCO-88 code 2230), step one resulted in medium level exposure to high-level disinfectants. In step two, for those nurses working in highly exposed units such as endoscopy, dialysis, intensive care unit, histology, pathology, pharmacology and operating rooms, exposures were re-assigned as high instead of medium.

**Table 2:** Occupation groups in the AWES-Asthma and Bhutan healthcare workers studies where exposures were re-assigned in step two according to OAsJEM instructions

ISCO-88 Code	ISCO-88 Description	Agent	Original level of exposure assigned	New exposure level assigned in step two	Number of participants who were assigned a new exposure		Instructions in the OAsJEM for re-assigning exposures in step two*
					**AWES-Asthma study**	**Bhutan healthcare workers study**	
2211	Biologists, botanists, zoologists and related professionals	Fish/shellfish	Medium	No	8	5	If there is no evidence of this person works regularly with fish recode fish = 0
2122	Pharmacologists, pathologists and related professionals	High level disinfectants	Medium	High	1	1	Recode chemical disinfectants = 2, if clear evidence that this person works in highly exposed units such as in histology, pathology, pharmacology, operating room, intensive care unit, endoscopy, dialysis
2221	Medical doctors	Latex	Medium	High	2	12	Recode latex = 2 if surgeon
2230	Nursing and midwifery professionals	Latex	High	No	14	0	Recode latex = 0 for nurses not based in hospitals (e.g. public health nurse, district nurse, nurse in school, occupational health nurse, research nurse, nurse educator etc.)
2230	Nursing and midwifery professionals	High level disinfectants	Medium	High	31	74	Recode chemical disinfectants = 2 if midwife or bronchoscopy, operating room or gastroenterology or geriatric or pediatric nurse or if clear evidence that this worker is using frequently such products (histology, pathology, pharmacology, intensive care unit, endoscopy, dialysis)
2230	Nursing and midwifery professionals	Drugs	No	High	2	10	Recode drugs = 2 only if oncology or geriatric nurse or if clear evidence that this worker is preparing drugs, keep 0 in other cases
3133	Medical equipment operators	High level disinfectants	Medium	High	10	7	Recode chemical disinfectants = 2 if radiography, histology, bronchoscopy, or gastroenterology technician, or if technician working in hospital sterile supply department; otherwise keep 1
3211	Life science technicians	High level disinfectants	No	High	2	19	Recode chemical disinfectants = 2 if clear evidence that this person works in histology, pathology, pharmacology otherwise keep 0
3221	Medical assistants	High level disinfectants	No	High	0	1	Recode chemical disinfectants = 2 if clear evidence that this person works in highly exposed units such as in histology, pathology, pharmacology, operating room, intensive care unit, endoscopy, dialysis
3225	Dental assistants	Acrylates	Medium	No	0	14	If working as a dental hygienist recode acrylate = 0
3228	Pharmaceutical assistants	Latex	Medium	No	1	0	Recode latex = 0 if clear evidence of no use of latex gloves
3228	Pharmaceutical assistants	Drugs	High	Medium	1	0	Recode drugs = 1 if not clear evidence that this worker is preparing drugs, keep 2 in other cases
3228	Pharmaceutical assistants	Amines	No	Medium	0	7	If clear evidence of daily use of drugs, recode amines = 1, otherwise leave as is
3229	Modern health associate professionals (except nursing) not elsewhere classified	High level disinfectants	No	High	0	8	Recode chemical disinfectants = 2 if clear evidence that this person works in in highly exposed units such as in histology, pathology, pharmacology, operating room, intensive care unit, endoscopy, dialysis
3229	Modern health associate professionals (except nursing) not elsewhere classified	Latex	No	High	0	8	Recode latex = 2 if clear evidence that this person works in personal nursing care in an operating room, a dental practice, or a geriatric care facility, otherwise leave as is
3231	Nursing associate professionals	Latex	High	No	1	0	Recode latex = 0 for associate nursing professionals not based in hospitals (e.g. public health, in school, occupational health nurse, research, education etc.)
5132	Institution-based personal care workers	Latex	Medium	High	0	18	Recode latex = 2 if clear evidence that this person works in personal nursing care in an operating room, oncology, a dental practice, or a geriatric care facility or in hospital, otherwise leave as is
5132	Institution-based personal care workers	High level disinfectants	Medium	High	0	18	Recode chemical disinfectant = 2 if clear evidence that this person works in personal nursing care in an operating room, oncology, a dental practice, or a geriatric care facility or in hospital, otherwise leave as is
5133	Home-based personal care workers	House dust mites	Medium	High	16	0	Recode house dust mite = 2 if evidence that a major part of the work is cleaning or sweeping in private home and mites are common in homes in the considered country
9132	Helpers and cleaners in offices, hotels and other establishments	High level disinfectants	Medium	High	9	16	Recode as chemical disinfectant = 2 if work is mainly indoor cleaning or extensive personal care of elderly persons in institution; otherwise leave as it is

For the AWES-Asthma study, step two resulted in exposures being re-assigned from high/medium to no exposure for 24 participants (for fish/shellfish and latex) and from no exposure to high exposure for four participants (for high-level disinfectants and drugs: Table 2). Exposures were re-assigned from medium to high for 51 participants (for latex, high-level disinfectants and house mites) and from high to medium for one participant (for drugs).

In the Bhutan healthcare workers study in step two, exposures were re-assigned from medium to no exposures for 19 participants (for fish/shellfish and acrylates) and from no exposures to medium/high exposures for 53 participants (for amines, drugs, latex, and high-level disinfectants). Exposures were re-assigned from medium to high for 146 participants (for latex and high-level disinfectants).

### Statistical analysis

In OccIDEAS, exposures were assigned as ‘no’ or ‘probable’ whereas the exposures in the OAsJEM were assigned as ‘high’, ‘medium’, and ‘no’ exposures. Therefore, a binary variable (exposed/unexposed) was created for exposures assessed by the JEM, where high and medium exposures were combined together and recoded as exposed.

All analyses were conducted using STATA 16 (StataCorp, College Station. TX). Frequencies were calculated to describe the prevalence of exposures to the various asthmagens assessed by OccIDEAS and the OAsJEM. Cohen’s Kappa coefficients (κ) and confidence intervals together with percentage agreement were estimated to analyse the agreement between the exposures assessed by the two methods. The agreement was interpreted using established cut-points for κ (i.e., poor 0.00-0.20, fair 0.21–0.40, moderate 0.41–0.60, strong 0.61–0.80, and almost perfect agreement 0.81-1.00) and percentages of agreement (≥ 75% was considered acceptable) [29]. In addition, for agents with lowest agreement between the two methods (for high-level disinfectants and acrylates), the proportion of participants assigned as exposed by their ISCO-88 codes was calculated for the two methods.

## Results

### The AWES-Asthma study

With both exposure assessment methods, there were high prevalences of exposure to cleaning and disinfecting agents (77.9% by OccIDEAS and 76.3% by the OAsJEM: Table 3) and latex (62.2% by OccIDEAS and 79.1% by the OAsJEM). The most striking difference in prevalence was seen for exposure to high-level disinfectants (4.9% in OccIDEAS versus 81.7% in the OAsJEM). For all other agents, the prevalence of exposures was quite low when assessed by either method. For a majority of agents, the prevalence was higher when assessed by OccIDEAS than when assessed by the OAsJEM. In addition, the exposure assessment by OccIDEAS identified exposures to a greater number of agents than that by the OAsJEM (16 versus 7). Of the eight additional agents identified by OccIDEAS, the ISCO-88 titles of the two agents with the highest prevalence that were assigned as exposed by OccIDEAS were as follows: (i) exposure to plants - nursing and midwifery professionals, nursing associate professionals, and personal care workers (institution and home-based); and (ii) exposure to bio-aerosols - biologists, botanists, zoologists and related professionals (e.g., microbiologists, bacteriologists) nursing and midwifery professionals, nursing associate professionals, personal care workers (institution and home-based), and helpers and cleaners.

**Table 3:** Comparison of OccIDEAS and the Asthma-specific JEM (OAsJEM) assessments of exposure to asthmagens among healthcare workers in Australia (N = 426)

	n (%) exposed		2 × 2				Cohen’s Kappa (95% confidence interval)	% agreement
	**OccIDEAS**	**OAsJEM**	**YY** ^a^	**YN** ^b^	**NY** ^c^	**NN** ^d^		
Cleaning/disinfecting agents	332 (77.9)	325 (76.3)	266	66	59	35	0.17 (0.07–0.27)	70.66
Latex	265 (62.2)	337 (79.1)	219	46	118	43	0.10 (0.01–0.19)	61.50
Arthropods/mites antigens	56 (13.2)	28 (6.6)	18	38	10	360	0.37 (0.24–0.51)	88.73
Acrylates	27 (6.3)	11 (2.6)	2	25	9	390	0.07 (-0.06–0.21)	92.02
High-level disinfectants	21 (4.9)	348 (81.7)	20	1	328	77	0.02 (0.00–0.03)	22.77
Derived from animals	11 (2.6)	8 (1.9)	5	6	3	412	0.52 (0.24–0.79)	97.89
Drugs	6 (1.4)	10 (2.4)	3	3	7	413	0.36 (0.06–0.67)	97.65
Plants	81 (19.0)	0 (0.0)	-	-	-	-	-	-
Bio-aerosols	57 (13.4)	0 (0.0)	-	-	-	-	-	-
Pesticides	51 (12.0)	0 (0.0)	-	-	-	-	-	-
Biological enzymes	45 (10.6)	0 (0.0)	-	-	-	-	-	-
Epoxy	4 (0.9)	0 (0.0)	-	-	-	-	-	-
Foods	3 (0.7)	0 (0.0)	-	-	-	-	-	-
Amines	2 (0.5)	0 (0.0)	-	-	-	-	-	-
Derived from fish	1 (0.2)	0 (0.0)	-	-	-	-	-	-
Isocyanates	1 (0.2)	0 (0.0)	-	-	-	-	-	-
Flour associated antigens	0 (0.0)	0 (0.0)	-	-	-	-	-	-
Metals	0 (0.0)	0 (0.0)	-	-	-	-	-	-
Wood dusts	0 (0.0)	0 (0.0)	-	-	-	-	-	-

^a^ Yes/Yes; ^b^ Yes/No; ^c^ No/Yes; ^d^ No/No with the first yes/no representing assessment by OccIDEAS

Agreement between the two methods could be assessed for seven agents (Table 3). Although the percentage agreement was acceptable for five of the seven agents (i.e., ≥ 75%), the agreement as indicated by κ for all agents was poor to fair (ranging from 0.02 to 0.37), except for exposure to agents derived from animals where the agreement was moderate (κ = 0.52).

Table 4 shows the proportion of participants assigned as exposed to high-level disinfectants and acrylates using the two methods. All participants (100%) in twelve ISCO-88 codes were assigned as exposed to high-level disinfectants by the OAsJEM; whereas in the OccIDEAS assessment, the proportion of participants who were exposed in the same ISCO-88 codes varied. Similarly, all participants (100%) in two ISCO-88 codes were assigned as exposed to acrylates by the OAsJEM, whereas in the OccIDEAS assessment, participants from four ISCO-88 codes were exposed and the proportions varied.

**Table 4:** Participants assigned as exposed to high-level disinfectants and acrylates in AWES-Asthma study by the OAsJEM and OccIDEAS

ISCO-88 code	ISCO-88 Title	Total participants in each ISCO-88 code	Exposure assessment by OAsJEM		Exposure assessment by OccIDEAS	
			**Exposed** **n**	**Exposed** **%**	**Exposed** **n**	**Exposed** **%**
**High-level disinfectants**						
2211	Biologists, botanists, zoologists and related professionals	8	8	100.0	6	75.0
2212	Pharmacologists, pathologists and related professionals	1	1	100.0	0	0.0
2221	Medical doctors	18	18	100.0	0	0.0
2222	Dentists	2	2	100.0	0	0.0
2230	Nursing and midwifery professionals	200	200	100.0	9	4.5
3133	Medical equipment operators	10	10	100.0	1	10.0
3211	Life science technicians	2	2	100.0	1	50.0
3225	Dental assistants	9	9	100.0	1	11.1
3231	Nursing associate professionals	23	23	100.0	1	4.3
3232	Midwifery associate professionals	1	1	100.0	0	0.0
5132	Institution-based personal care workers	60	60	100.0	1	1.7
5133	Home-based personal care workers	28	0	0.0	1	3.6
9132	Helpers and cleaners in offices, hotels and other establishment	14	14	100.0	0	0.0
**Acrylates**						
2221	Medical doctors	18	0	0.0	8	44.4
2222	Dentists	2	2	100.0	0	0.0
2230	Nursing and midwifery professionals	200	0	0.0	16	8.0
3225	Dental assistants	9	9	100.0	2	22.2
5132	Institution-based personal care workers	60	0	0.0	1	1.7

### The Bhutan healthcare workers study

In the Bhutan study, only nine of the 19 agents were assessed. The prevalence of exposure assessed by OccIDEAS was higher for four agents (latex, cleaning and disinfecting agents, acrylates, and epoxy) and the prevalence assessed by the OAsJEM was higher for three agents (high-level disinfectants, drugs, and amines: Table 5). In this study, as in the Australian study, exposure to latex and cleaning and disinfecting was high by both methods and the greatest difference in prevalence was seen for exposure to high-level disinfectants (35.1% in OccIDEAS versus 89.2% in the OAsJEM). Exposure assessment by the OAsJEM identified a slightly higher number of agents than by OccIDEAS assessment (6 versus 5).

**Table 5:** Comparison of OccIDEAS and the Asthma-specific JEM (OAsJEM) assessments of exposure to asthmagens among healthcare workers in Bhutan (N = 370)

	n (%) exposed		2 × 2				Cohen’s Kappa (95% confidence interval)	% agreement
	**OccIDEAS**	**OAsJEM**	**YY** ^a^	**YN** ^b^	**NY** ^c^	**NN** ^d^		
Latex	350 (94.6)	293 (79.2)	281	69	12	8	0.09 (-0.01–0.18)	78.11
Cleaning/ disinfecting agents	318 (86.0)	213 (57.6)	194	124	19	33	0.13 (0.05–0.21)	61.35
High-level disinfectants	130 (35.1)	330 (89.2)	121	9	209	31	0.04 (0.00–0.09)	41.08
Acrylates	40 (10.8)	14 (3.8)	0	40	14	316	-0.06 (-0.09 - -0.03)	85.41
Epoxy	3 (0.8)	0 (0.0)	-	-	-	-	-	-
Drugs	0 (0.0)	21 (5.7)	-	-	-	-	-	-
Amines	0 (0.0)	7 (1.9)	-	-	-	-	-	-
Bio-aerosols	0 (0.0)	0 (0.0)	-	-	-	-	-	-
Isocyanates	0 (0.0)	0 (0.0)	-	-	-	-	-	-

^a^ Yes/Yes; ^b^ Yes/No; ^c^ No/Yes; ^d^ No/No with the first yes/no representing assessment by OccIDEAS

Agreement between the two methods could be assessed for four agents (Table 5). Agreement as indicated by κ was poor for all the four agents (ranging from − 0.06 to 0.13), although the percentage agreement was acceptable for two agents (i.e., ≥ 75% for latex and acrylates).

Table 6 shows the proportion of participants assigned as exposed to high-level disinfectants and acrylates using the two methods. All participants (100%) in ten ISCO-88 codes were assigned as exposed to high-level disinfectants by the OAsJEM, whereas in the OccIDEAS assessment, the proportion of participants who were exposed in the same ISCO-88 codes varied. All dentists and dental assistants who were not dental hygienists were assigned as exposed to acrylates by the OAsJEM, whereas in the OccIDEAS assessment, participants from six ISCO-88 codes were exposed to acrylates and the proportions varied.

**Table 6:** Participants assigned as exposed to high-level disinfectants and acrylates in the Bhutan healthcare workers study by the OAsJEM and OccIDEAS

ISCO-88 code	ISCO-88 Title	Total participants in each ISCO-88 code	Exposure assessment by OAsJEM		Exposure assessment by OccIDEAS	
			**Exposed** **n**	**Exposed** **%**	**Exposed** **n**	**Exposed** **%**
**High-level disinfectants**						
2211	Biologists, botanists, zoologists and related professionals	5	5	100.0	4	80.0
2212	Pharmacologists, pathologists and related professionals	1	1	100.0	1	100.0
2221	Medical doctors	42	42	100.0	3	7.1
2222	Dentists	13	13	100.0	8	61.5
2224	Pharmacists	3	0	0.0	1	33.3
2230	Nursing and midwifery professionals	176	176	100.0	54	30.7
3133	Medical equipment operators	7	7	100.0	1	14.3
3211	Life science technicians	32	19	59.4	22	68.8
3221	Medical assistants	10	10	100.0	2	20.0
3225	Dental assistants	15	15	100.0	15	100.0
3226	Physiotherapist and related associated professionals	5	0	0.0	1	20.0
3229	Modern health associate professionals (except nursing) not elsewhere classified	9	8	88.9	5	55.6
5132	Institution-based personal care workers	18	18	100.0	9	50.0
8322	Car, taxi and van drivers	4	0	0.0	1	12.5
9132	Helpers and cleaners in offices, hotels and other establishment	16	16	100.0	3	18.8
**Acrylates**						
2221	Medical doctors	42	0	0.0	4	9.5
2222	Dentists	13	13	100.0	0	0.0
2230	Nursing and midwifery professionals	176	0	0.0	30	17.0
3225	Dental assistants	15	1	6.7	1	6.7
3226	Physiotherapist and related associated professionals	5	0	0.0	1	20.0
3229	Modern health associate professionals (except nursing) not elsewhere classified	9	0	0.0	3	33.3
9132	Helpers and cleaners in offices, hotels and other establishment	16	0	0.0	1	6.3

## Discussion

This study aimed to compare exposure assessment of asthmagens in healthcare workers by a task-specific algorithm-based method (OccIDEAS) and a job-specific matrix method (OAsJEM) using data from two separate studies conducted in Australia and Bhutan to determine an appropriate assessment method for use in LMICs for future research. The agreement between the two methods as assessed by Cohen’s κ was poor to fair for most of the agents assessed in the AWES-Asthma study and was poor for all the agents assessed in the Bhutan healthcare workers study.

The poor agreement between the two methods appears to be mainly due to the inter-individual variation of tasks within a job. OccIDEAS consists of task-based questions to which expert-derived algorithms are applied for assigning exposures, whereas in the OAsJEM the same exposure is assigned to a job title irrespective of inter-individual variations. This was shown when the proportion of participants assigned as exposed was compared between the two methods for the agents with the lowest agreement (for high-level disinfectants and acrylates). For exposure assessment to high-level disinfectants in the OAsJEM, all participants in twelve and ten ISCO-88 codes were assigned as exposed in the AWES-Asthma and the Bhutan healthcare workers studies, respectively, resulting in an overestimation of exposure. When exposures were assessed by OccIDEAS, the proportion of participants in each ISCO-88 codes varied considerably. In OccIDEAS, participants were asked specifically if they carried out sterilisation or instrument disinfection, and if so, they were asked to select the chemical they used from a list of chemicals (glutaraldehyde, formaldehyde, ethylene oxide, quaternary ammonium compounds, peracetic acid, or other chemicals they could volunteer). For exposure to acrylates, exposure assessment by OccIDEAS assigned exposures to varying proportions of participants from four to six different ISCO-88 groups, whereas only participants who were dentists or dental assistants (but were not dental hygienists) were assigned as exposed to acrylates by the OAsJEM resulting in an underestimation of exposure. For acrylate exposure in OccIDEAS, participants were asked if they handled bone cement, used cyanoacrylate super glues, or whether they manufactured crowns, false teeth or bridges and if they used an enclosed system to do so. Rule-based algorithms principally aim to increase inter-individual contrasts in exposures using task-based determinants of exposures [5]. Task-specific questions are not only less prone to recall bias as participants are able to report work tasks more accurately [1], but, as shown in this study, they also assist in identifying within-job differences in exposure. The findings of this study are similar to the only other study that has evaluated agreement between a task-based questionnaire algorithm and a JEM, where similar low levels of agreement between the two methods was reported in the assessment of exposure to asbestos (κ = 0.36 and weighted κ = 0.26) [30].

The level of agreement between the two methods was poor for all four agents that could be assessed in both the Australian and Bhutanese studies (i.e., cleaning and disinfecting agents, latex, acrylates and high-level disinfectants), with most of the κ values lower in the Bhutanese study. In addition to task-based inter-individual variations, the poor agreement between the two methods could also be due to inter-country differences in exposure circumstances such as work environments and available control measures. Exposure circumstances were taken into account in OccIDEAS. The task-based questions in OccIDEAS for the AWES-Asthma study were developed for the Australian workplaces and included the use of various control measures where appropriate [14]. Similarly, the questionnaire used in the Bhutan healthcare workers study was adapted to suit the Bhutanese work environment [6]. The findings from this study raise the issue of the applicability of a JEM developed for one country to exposure circumstances in another country. Previous studies have shown differences in agreement between JEMs developed in different countries. In a study comparing the SK-JEM (developed in France) to a JEM developed for use primarily in Northern Europe (N-JEM), the kappa score for asthma-related exposure was 0.78 [31]. However, when the N-JEM was compared to a JEM developed in the US (USA-JEM), the kappa score was 0.54 [32]. Other studies have reported some misclassification of exposures in the application of JEMs outside the country of origin or geographical region, and reported that this misclassification depended on the agents assessed and their prevalence of exposures [33, 34]. As indicated by the very low levels of agreement between the two methods in the Bhutan healthcare workers study, the issue of cross-country applicability of JEMs is of particular significance when applying a JEM from a high-income country to a low-income country where the exposure circumstances may be completely different.

OccIDEAS identified exposures to a greater number of agents than the JEM in the present study. This included agents that are not typically associated with healthcare workers, and hence were not detected by the JEM, such as plants, bioaerosols, pesticides and biological enzymes. This finding provides additional support for the advantage of task-based exposure assessment over using job title as a surrogate measure of exposure.

The expert assessment method is considered the best available method for exposure assessment and is usually used as a gold standard for comparison [1, 3]. Although comparisons were not made with the expert assessment method in this study, a previous study assessing the agreement between OccIDEAS and expert assessment showed moderate to almost perfect agreement in assessing exposures to 10 common asthmagens [35]. Other studies that have compared rule-based exposure assessment methods using task level data to expert assessments have shown reasonable agreements for agents such diesel exhausts, pesticides, solvents and asbestos [30, 36, 37]. On the other hand, studies comparing inter-method agreement between JEMs and experts have largely shown poor to fair agreement for various agents such as asbestos (κ = 0.10 to 0.36), silica (κ = 0.38), solvents (κ = 0.07 to 0.28), lead (κ = 0.33), insecticides (κ = 0.46) and polycyclic aromatic hydrocarbons (κ = 0.40) [30, 34, 38, 39].

This study has some limitations. Since OccIDEAS assessed exposures as yes/no, the semi-quantitative metrics of the OAsJEM were recoded to correspond to that of OccIDEAS. The agreement was therefore assessed for yes/no and not for intensity. Further, because of small numbers of healthcare workers exposed to some agents, the agreement could not be evaluated for all the agents, and exposures to only a small number of agents could be compared (especially for the Bhutan healthcare workers study). In addition, there could have been some discrepancies when grouping the agents for comparison, which may have affected the exposure metrics assigned to these grouped agents. This might have occurred when grouping the agents for cleaning and sterilising agents and for high-level disinfectants since these groups comprise a wide variety of chemicals. Despite these limitations, this is one of the few studies that has assessed the agreement between rule-based automatic algorithms and a JEM and is the first study to do so for asthmagen exposures. This study is also the first study to include comparison of exposure assessment in a low-income country.

## Conclusion

There was poor to fair agreement in the assessment of exposure to asthmagens in healthcare workers between a task-specific algorithm-based method (OccIDEAS) and a job-specific matrix method (OAsJEM), which could be due to differences in inter-individual variations of tasks within a job. The OAsJEM overestimated exposures to high-level disinfectants and acrylates. OccIDEAS identified exposures to more agents than the OAsJEM, including agents that were not typically associated with healthcare workers. OccIDEAS gathers individually tailored data about current tasks so is more likely to identify differences in exposures between countries and over time than the OAsJEM. As compared to the OAsJEM, OccIDEAS appeared to be more appropriate for evaluating cross-country exposures to asthmagens in healthcare workers due to its inherent quality of assessing task-based determinants and its versatility in being adaptable for use in different countries with different exposure circumstances.

## Data Availability

All the data supporting the findings have been presented in the manuscript; the datasets used and/or analysed during the current study are available from the corresponding author on reasonable request.
